#  High-Throughput Sequencing of Circulating MicroRNAs in Plasma and Serum during Pregnancy Progression

**DOI:** 10.3390/life11101055

**Published:** 2021-10-08

**Authors:** Elena S. Vashukova, Polina Y. Kozyulina, Roman A. Illarionov, Natalya O. Yurkina, Olga V. Pachuliia, Mariya G. Butenko, Tatyana B. Postnikova, Lada A. Ivanova, Dina R. Eremeeva, Marina S. Zainulina, Olesya N. Bespalova, Andrey S. Glotov

**Affiliations:** 1 Department of Genomic Medicine, D.O. Ott Research Institute for Obstetrics, Gynecology and Reproduction, 199034 St. Petersburg, Russia; vi_lena@list.ru (E.S.V.); polykoz@gmail.com (P.Y.K.); r.a.illarionov@gmail.com (R.A.I.); yurkina.natali1002@gmail.com (N.O.Y.); for.olga.kosyakova@gmail.com (O.V.P.); butenkomariabutenko@gmail.com (M.G.B.); ptb20@mail.ru (T.B.P.); shiggerra@mail.ru (O.N.B.); 2 Signal Regulation Laboratory, All-Russia Research Institute for Agricultural Microbiology, 196608 St. Petersburg, Russia; 3 Institute of Translational Biomedicine, St. Petersburg State University, 199034 St. Petersburg, Russia; 4 Department of Chemical and Biotechnology, St. Petersburg State Institute of Technology, Technical University, 190013 St. Petersburg, Russia; 5 Antenatal Clinic No.26 Maternity Hospital No.10, 198259 St. Petersburg, Russia; roddom10@zdrav.spb.ru; 6 II Obstetric Department Pathology of Pregnancy, V. F. Snegirev Maternity Hospital No.6, 192014 St. Petersburg, Russia; dina-bikmullina@yandex.ru (D.R.E.); zainulina@yandex.ru (M.S.Z.)

**Keywords:** microRNA, pregnancy, plasma, serum, high-throughput sequencing

## Abstract

Although circulating microRNAs (miRNAs) in maternal blood may play an important role in regulation of pregnancy progression and serve as non-invasive biomarkers for different gestation complications, little is known about their profile in blood during normally developing pregnancy. In this study we evaluated the miRNA profiles in paired plasma and serum samples from pregnant women without health or gestational abnormalities at three time points using high-throughput sequencing technology. Sequencing revealed that the percentage of miRNA reads in plasma and serum decreased by a third compared to first and second trimesters. We found two miRNAs in plasma (hsa-miR-7853-5p and hsa-miR-200c-3p) and 10 miRNAs in serum (hsa-miR-203a-5p, hsa-miR-495-3p, hsa-miR-4435, hsa-miR-340-5p, hsa-miR-4417, hsa-miR-1266-5p, hsa-miR-4494, hsa-miR-134-3p, hsa-miR-5008-5p, and hsa-miR-6756-5p), that exhibit level changes during pregnancy (*p*-value adjusted < 0.05). In addition, we observed differences for 36 miRNAs between plasma and serum (*p*-value adjusted < 0.05), which should be taken into consideration when comparing the results between studies performed using different biosample types. The results were verified by analysis of three miRNAs using qRT-PCR (*p* < 0.05). The present study confirms that the circulating miRNA profile in blood changes during gestation. Our results set the basis for further investigation of molecular mechanisms, involved in regulation of pregnancy, and the search for biomarkers of gestation abnormalities.

## 1. Introduction

MiRNAs are a class of noncoding single-stranded small RNAs approximately 21–25-nucleotides in length [1,2,3]. miRNAs negatively regulate gene expression by binding to the 3′-untranslated region of the target mRNAs and thus may possess important control functions in diverse biological processes, including embryonic development, cellular differentiation, proliferation, and apoptosis [1,2,3]. MiRNAs are shown to be involved in regulation of pregnancy progression [4]. Differentially expressed miRNAs have been detected in the placenta at different gestational ages and in pregnancy complications in numerous studies [1,4,5,6,7,8].

Biomarkers found in biological fluids are useful to study physio-pathological processes and to define actual disease progression [9]. MiRNAs can play an important role as one of these biomarker types. MiRNAs are stable in the extracellular environment, including in adverse conditions, and can be quantified using routine laboratory methods [10,11]. To date, some circulating miRNAs have already been established as prospective biomarkers of various diseases [12,13,14,15]. In recent years, intense research has focused on circulating miRNAs in the blood of pregnant women. However, the profile of circulating miRNAs in normal pregnancy remains poorly investigated.

There is a large number of independent studies searching for the association between circulating miRNAs in blood and common gestational disorders [1,5]. However, the results of these works are inconsistent. Variation in the miRNA profile could be explained by different study designs, in particular using different sample types [5]. Most commonly, plasma or serum is used for miRNA analysis, but the choice between these two biofluids for miRNA detection is still a focus of debate. The differences in miRNA concentrations and profiles between plasma and serum have been demonstrated by some authors [15,16], whereas others have shown opposite results [10,13]. The mixed results were obtained when comparing plasma and serum samples from healthy pregnant women [17,18]. There is only one study comparing the difference in miRNA content between serum and plasma from pregnant women using high-throughput sequencing [17]. However, this study analyzed only three paired serum and plasma samples and these samples were pooled prior to library preparation, which reduced the statistical reliability of the sequencing results. In addition, the samples were taken from women at one gestational age (second trimester) [17].

There is evidence that the blood miRNA profile changes with pregnancy progression, but the data on the content of miRNAs in blood during gestation progression is fragmented. Most studies detect the circulating miRNAs at a single time point during gestation [1,2,3]. There are some that included analysis of the circulating miRNA spectrum during progression of the normal pregnancy, however a part of the analysis was limited to quantitative PCR and microarray [19,20]. Only a few studies have used high-throughput sequencing platforms [6,21,22]. There is only one study where plasma miRNA profiles at different pregnancy stages (first, second, and third trimesters) were analyzed, but plasma samples were collected from different pregnant women during gestation progression and samples of each time point were pooled prior to library preparation [21]. In another study the miRNA profiles in plasma were characterized during early pregnancy between 6–10- and 11–23-weeks’ gestation [6]. In addition, the changes in exosomal miRNAs circulating in the plasma across gestation (first, second, and third trimesters and at the time of delivery) were described [22]. Notably, these studies used plasma, while miRNA profiles in serum across pregnancy are not yet described by high-throughput sequencing. The study designs varied and the data reported were insufficient to produce accurate representation of changes in levels of miRNA during normal pregnancy.

This study aimed to evaluate the miRNA profiles in paired plasma and serum samples from pregnant women without obstetric and gynecological abnormalities at three gestational ages (first, second, and third trimesters) using the technology of high-throughput sequencing. This is important for enhancing knowledge of circulating miRNA content during gestation and understanding their role in the regulation of pregnancy processes.

## 2. Results

### 2.1. Characteristics of Subjects

The characteristics of the pregnant women included in the study, the gestational ages of sampling, and the pregnancy outcomes are presented in Table 1. The research group included three primiparous and four multiparous women. The latter had no gestational abnormalities in previous pregnancies.

### 2.2. Small RNA Sequencing Results

We determined the profiles of small RNAs in the blood plasma and serum samples of pregnant women (n = 7) at three time points of gestation using the technology of high-throughput sequencing. For this purpose, 42 small RNA libraries were prepared—21 libraries for each type of biofluid.

**Figure 1:**
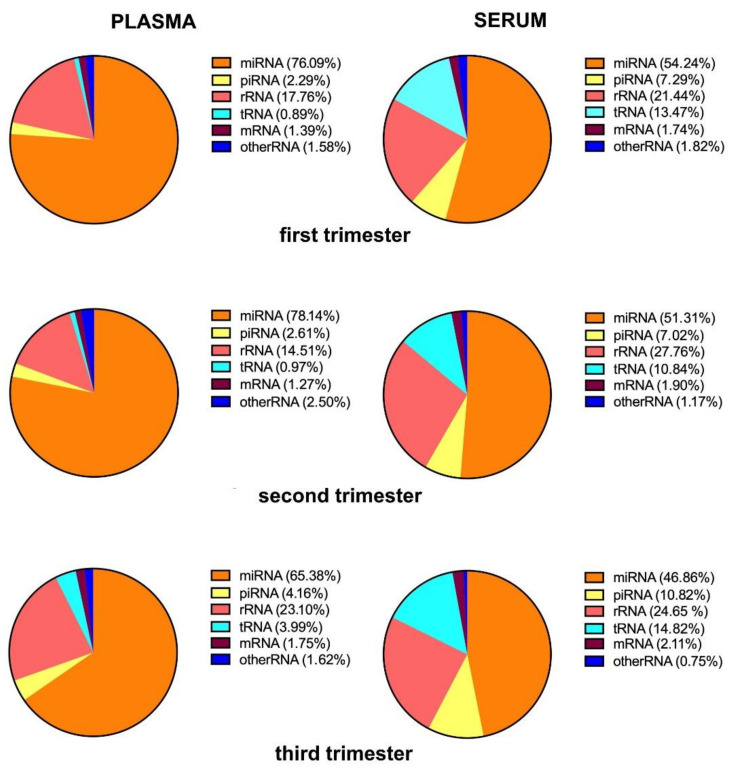
RNA categories in plasma and serum of pregnant women according to the number of high-throughput sequencing reads.

The total reads obtained from plasma and serum samples were 34,013,380 and 32,981,722 reads in the first, 38,145,207 and 34,342,265 in the second, and 38,391,138 and 33,991,358 in the third trimester, respectively. The percentage of mappable to the human genome (hg19) reads were 46.7%, 50.7%, and 42.5% for plasma and 45.5%, 41.3%, and 44.1% for serum in the first, second, and third trimesters, respectively. MiRNAs were the largest fraction of all small RNA types at the different pregnancy stages in both fluids (Figure 1). A small percentage of the mapped reads corresponded to other human non-coding RNAs, including rRNA, tRNA, piRNA, mRNA, and other RNA (Figure 1).

However, the distributions of RNA categories were different between plasma and serum samples (Figure 1). The percentages of mapped reads corresponding to miRNAs were higher for plasma compared to serum at all gestational ages (Figure 1). At the same time serum samples contained more reads that mapped to tRNAs and piRNAs (Figure 1). In addition, the relative proportions of the RNA categories differed across pregnancy in both fluids. The percentage of miRNA was within the range of 65.4% to 78.1% for plasma and 46.9% to 54.2% for serum, with a minimal miRNA fraction detected at the third trimester in both biofluids. In contrast, the sequencing fragments of tRNAs and piRNAs were more frequently detected at the third (4.0% and 4.2% in plasma; 14.8% and 10.8% in serum, respectively), as compared with the first (0.9% and 2.3% in plasma; 13.5% and 7.3% in serum, respectively), and the second (1.0% and 2.6% in plasma; 10.8% and 7.0% in serum, respectively), trimesters.

### 2.3. Plasma and Serum miRNA Profiles in Different Stages of Pregnancy

**Figure 2:**
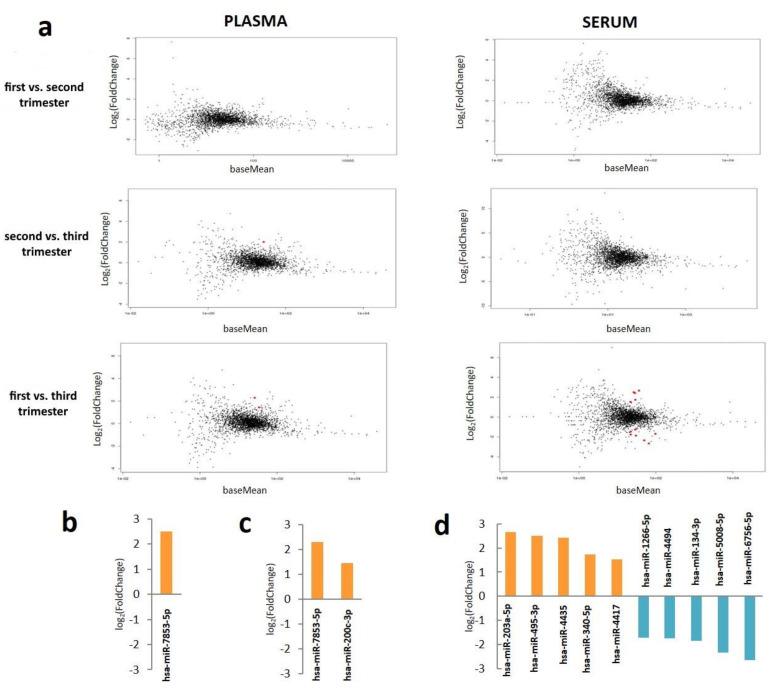
miRNAs that significantly change across gestation in plasma and serum taken from women without pregnancy complications. (**a**) MA plot comparisons of miRNA content in plasma and serum across three gestational ages. MA analysis revealed levels of one miRNA changed between first and second trimesters, two miRNAs between first and third trimesters for plasma and ten miRNAs between first and third trimesters for serum (*p*-value adjusted < 0.05). miRNAs that changed significantly are highlighted in red. (**b**) miRNAs that changed significantly between second and third trimesters, (**c**) miRNAs that changed significantly between first and third trimesters in plasma, and (**d**) miRNAs that changed significantly between first and third trimesters in serum. Upregulated and downregulated miRNA in third trimester compared to first or second trimesters are indicated by upward and downward bars, respectively. Standard errors are indicated by error bars.

In total, 2299 known miRNAs were detected in plasma samples taken at the first, 2339 miRNAs at the second, and 2328 miRNAs at the third trimester, respectively. The top ten miRNAs with the highest level in plasma were common across all gestational ages and included hsa-miR-16-5p, hsa-miR-486-5p, hsa-let-7b-5p, hsa-miR-486-3p, hsa-miR-92a-3p, hsa-let-7a-5p, hsa-let-7i-5p, hsa-miR-122-5p, hsa-miR-423-5p, and hsa-let-7f-5p. Among all miRNAs identified we found two miRNAs, which exhibited statistically significant level changes during pregnancy (Figure 2a). The level of the miRNA hsa-miR-7853-5p increased in the third as compared with the first and second trimesters (Figure 2b,c, respectively). The level of the miRNA hsa-miR-200c-3p was higher at third as compared with the first trimester (Figure 2c). We did not observe statistically significant differences in the levels of all the other identified miRNAs between the first and the second trimesters (*p* > 0.05 for each miRNA) (Figure 2a)

In total, 2077 miRNAs from serum samples were sequenced in the first, 2294 miRNAs in the second, and 2200 miRNAs in the third trimesters, respectively. The top ten miRNAs with the highest level in serum differed between gestational ages. They included eight miRNAs detected at all stages of pregnancy (hsa-miR-16-5p, hsa-miR-486-5p, hsa-let-7b-5p, hsa-miR-486-3p, hsa-miR-92a-3p, hsa-let-7a-5p, hsa-let-7i-5p, and hsa-miR-122-5p), one in the first and second trimesters (hsa-miR-423-5p), one in the second and third trimesters (hsa-let-7f-5p), and two miRNAs only in the first (hsa-miR-223-3p) and second (hsa-miR-6083) trimesters. The level of all identified miRNAs did not change between the first and second, and the second and the third trimesters (*p* > 0.05 for each miRNA) (Figure 2a). However, significant differences were identified between the first and the third trimesters (Figure 2a). In total the levels of ten miRNAs were different between the first and third trimesters. Among them, five were upregulated and five were downregulated in the first trimester when compared to the third trimester. These miRNAs are shown in Figure 2d.

The full list of miRNAs identified and profiled in plasma and serum is presented in Additional Files 1 (Tables S1–S3 in supplementary) and 2 (Tables S4–S6), respectively. 

### 2.4. Comparison of miRNA Profiles between Plasma and Serum

We performed the comparative analysis of different miRNA types between paired serum and plasma samples. The analysis was performed on all 42 samples. In order to compensate for variance between trimesters the additional trimester factor was included into the DeSeq2 matrix design thus allowing the application of variance batch compensation rather than excluding samples. In total, 36 miRNAs exhibited altered level patterns in plasma, when compared with those in serum. Fifteen miRNAs were detected at higher and 21 miRNAs at lower levels, in plasma as compared with the serum (Table 2). 

The analysis was performed on samples from all three trimesters (21 samples for serum and 21 samples for plasma). In order to compensate for variance between trimesters the additional trimester factor was included into DeSeq2 matrix design thus allowing the application of variance batch compensation rather than excluding samples.

According to miRDB target prediction database, a total of 4239 genes can be potential targets of these miRNAs. Gene enrichment analysis of the predicted target genes showed that there were 40 GO term gene sets and 117 KEGG pathways that were significantly overrepresented in the list of potential miRNA targets. Table 3 and Table 4 exhibit the top 15 enriched GO terms and KEGG pathways across these miRNAs, respectively. The majority of the significant GO terms were associated with the regulation of transcription. There were also a number of significantly increased GO categories, including protein kinase activity, GTPase activity and binding, ubiquitin-like protein transferase activity, SMAD, GDP, chromatin, beta-catenin cell adhesion molecule, phosphatidylinositol, and purine ribonucleoside binding (Table 3). KEGG enrichment analysis demonstrated that the majority of the differentially expressed miRNAs targeted genes involved in different signaling pathways (PI3K-Akt, MAPK, Ras, Rap1, calcium, and cAMP signaling pathways) and infections (papillomavirus, cytomegalovirus, T-cell leukemia virus 1, and shigellosis infection) (Table 4).

### 2.5. qRT-PCR Verification of High-Throughput Sequencing Data

**Figure 3:**
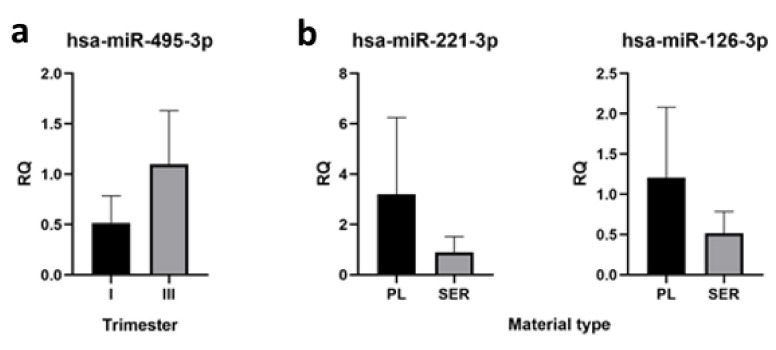
Verification of high-throughput sequencing data by qRT-PCR. (**a**) qRT-PCR verification of the change in the hsa-miR-495-3p level identified through high-throughput sequencing across the pregnancy gestation. (**b**) qRT-PCR validation of the changes in the hsa-miR-221-3p and hsa-miR-126-3p levels identified through high-throughput sequencing in plasma and serum. Standard errors are indicated by error bars. In all cases *p* < 0.05. RQ, relative target quantity; PL, plasma; SER, serum.

In order to provide additional evidence for the reliability of the high-throughput sequencing data, we repeated the analysis of three miRNAs (hsa-miR-221-3p, hsa-miR-126-3p, and hsa-miR-495-3p) with significant change (log2 (fold change) > 1.50) using qRT-PCR (Figure 3). The qRT-PCR results were consistent with high-throughput sequencing data—the serum level of miR-495-3p increased in the third trimester when compared to the first trimester (*p* < 0.05) (Figure 3a); the levels of miR-221-3p and miR-126-3p were increased in plasma samples relative to serum (*p* < 0.05) (Figure 3b).

## 3. Discussion

To date, there is little information about the circulating miRNA profiles in blood during healthy pregnancy. In the present study the miRNA profiles were detected and compared between three gestational ages in both plasma and serum samples from pregnant women without gestational complications using the high-throughput sequencing technology, one of the most effective and sensitive methods for detecting circulating miRNA spectrum today [23].

When evaluating the small RNA type distribution, we found that miRNA concentration in plasma and serum changed across the pregnancy. The percentage of miRNA mapping reads decreased in the third as compared with the first and the second trimesters in both fluids. The same tendency for plasma samples was described in the manuscript of Li et al. [21]. The authors analyzed small RNA profiles in plasma of healthy pregnant women using solid technology and found that the percentage of miRNA in plasma among other small RNAs was lowest in the third trimesters (21.62%) as compared with the first and second trimesters (43.48% and 52.04%, respectively) [21]. The decrease in circulating blood miRNAs level in the third trimester may be explained by the physiological changes that occur in pregnancy, which affect every organ system [24]. Most of these changes accumulate by the third trimester. The major changes are reflected in the blood plasma and serum composition [24]. They include changes in level of blood cells, coagulation factors, anticoagulants, lipids, hormones, ions, proteins, and other components [24], which probably affects the level of nucleic acids circulated in blood.

In our experiments, in total more than two hundred known miRNAs were detected from plasma and serum samples. This is in accordance with the miRNA number published [6,21,22]. The list of the top 10 highest level miRNAs is consistent with the data of other authors studying the microRNA profile in blood of healthy volunteers [23]. Of note, 8 of the 10 most abundant miRNAs detected were common in plasma and serum across all gestational ages.

The comparative analysis of miRNA levels between different pregnancy ages showed that the profiles of miRNAs in the third trimester differed from those in the first trimester in both fluids. We found two miRNAs (hsa-miR-7853-5p and hsa-miR-200c-3p) that were significantly upregulated in plasma in the third versus first trimester. Ten miRNAs showed statistically significant differences between first and third trimesters in serum. Among them, five miRNAs (hsa-miR-203a-5p, hsa-miR-495-3p, hsa-miR-4435, hsa-miR-340-5p, and hsa-miR-4417) were upregulated and five miRNAs (hsa-miR-1266-5p, hsa-miR-4494, hsa-miR-134-3p, hsa-miR-5008-5p, and hsa-miR-6756-5p) were downregulated. In addition, we observed an increase of hsa-miR-7853-5p levels in plasma in the third as compared with the first trimester. It may be proposed that these changes in miRNA levels may be functionally important for gestation progression and further pregnancy outcomes.

The biological role of deregulated microRNAs has not yet been accurately established. Some of them have been reported to potentially play an important role in various physiologic and pathologic processes—regulation of proliferation, migration, invasion, epithelial to mesenchymal transition, and apoptosis in cervical cancer (miR-203a-5p) [25,26], apoptosis in human prostate cancer cells (miR-1266-5p) [27], proliferation, cell cycle control, apoptosis, anoikis, invasion, and metastasis of different cancer types (miR-200c-3p) [28], carcinogenesis in the breast (miR-4435) [27] and ovary (miR-134-3p) [29], and development, immune responses, neovascularization, and carcinogenesis (miR-495-3p) [30].

Some of the detected miRNAs were previously associated with pregnancy. Thus, it has been reported that a number of miRNAs may be involved in the regulation of various functions in the placenta during pregnancy. Two miRNAs, miR-495-3p and miR-134-3p, are from the C14MC miRNA cluster. This is a region of chromosome 14 which encodes 52 miRNAs. Some of them are produced by the placenta in substantial quantities and an enter maternal circulation [31]. C14MC is exclusively expressed from the maternal allele. The precise biological function of the C14MC cluster in regulation of pregnancy processes is unknown. Presumably C14MC miRNAs are involved in embryonic development and neonatal metabolic adaptation, and deregulation of these miRNAs might lead to an abnormal pregnancy [4]. Also, it was shown that miR-495-3p is able to arrest proliferation, inhibit invasion, and induce the apoptosis in different cell types and it plays an essential role in inflammatory reactions and angiogenesis [29], which are the key physiological processes during pregnancy. miR-200c belongs to the miR-200 family, which is involved in placental function, in particular in the cellular transition from epithelial to mesenchymal phenotype which is a presupposition for extravillous trophoblast invasion of the maternal decidua [6]. miR-200c also has been reported to target mRNA of endothelial growth factor (*VEGF*) in trophoblasts, and therefore may exert effects on angiogenesis and vasculogenesis in placenta [32]. 

Notably, the four miRNAs that have changed levels during pregnancy in our study are associated with different pregnancy complications. miR-200c-3p displays an altered expression in placentas from pregnancies complicated by preeclampsia, preterm labor, and fetal macrosomia [33,34,35]. In addition, miR-200c-3p is downregulated at the first trimester in serum of pregnant women undergoing spontaneous abortion [36]. The level of miR-495-3p is deregulated in placenta from patients with preeclampsia and preterm labor [34], is decreased in the plasma and extracellular vesicles in second and third trimesters, but increased in the whole blood in the second trimester in women undergoing preterm labor [31,37]. Deregulated placental expression of miR-4494 is associated with preterm labor [38]. miRNA-340-5p is elevated in the whole blood cells of patients with gestational diabetes in the third trimester [39] and preterm delivery in the first trimester [40]. These data indicate the crucial role of these four miRNAs in regulation of pregnancy.

The levels of two miRNAs with significant differences have been previously reported to be changed in blood circulation during gestation without complications. Li et al. [20] observed that the level of miR-200c-3p changes in blood plasma across the normal pregnancy which is supported by our own findings. The miR-495-3p was found to significantly change in plasma exosomes across gestation [22].

Changes in levels of 10 miRNAs in the plasma or serum samples from pregnant women across gestation were revealed in this study for the first time. The functional role of these microRNAs in pregnancy needs to be clarified, which is the goal of further studies.

The significant differences in miRNA profiles between plasma and serum have been demonstrated in several studies [15,16,17,41], so we also compared miRNA content in these biofluids and found that miRNA mapping reads were enriched in plasma compared to serum at all gestational ages. This result is consistent with the work of other authors, who showed that the percentage of miRNA reads is higher in plasma compared to serum when using different methodologies of RNA extraction, library preparation, and sequencing [17,26,42]. This fact is consistent with the suggestion that plasma may be the more useful biofluid for circulating miRNA analysis [26,42], while serum needs more total sequencing reads per sample, and therefore is more expensive. On the other hand, for miRNA in serum we obtained more diverse results, which may be more useful in future for calculating models for pregnancy complication prediction. The lower miRNA level is thought to be a consequence of the coagulation process, occurring during serum collection [42]. Degradation of circulating miRNAs could happen in serum samples because of the RNases released from blood cells. Also, miRNAs could be adsorbed onto the blood clot during coagulation. At the same time cell lysis during the coagulation process could lead to the release of other RNA fragments [42]. In addition, we found statistically significant differences for 36 miRNAs between plasma and serum. mRNAs of many genes are potential targets of these miRNAs. Pathway analysis revealed that these miRNAs are associated with a variety of processes, which are not specific to any normal or pathologic conditions, which should be taken into consideration when comparing the results between studies performed using different biosample types. Differences in miRNA profiles in plasma and serum may explain the inconsistent results between different works and demonstrate that results from studies devoted to association of miRNAs with various physiologic and pathologic processes in plasma and serum should not be directly compared.

The limitations of our study are the relatively small sample selection for high-throughput sequencing. However, the experimental approach was based on usage of a protocol to reduce possible sources of variability. All samples were carefully selected for full term singleton pregnancies, non-complicated by maternal health conditions and gestational abnormalities. The paired samples of plasma and serum were collected from the same pregnant women across the gestation. The samples were obtained, processed, and stored at the same time. Common protocols of RNA extraction and library preparation were used. All libraries were sequenced in the same run. In order to provide additional evidence for the reliability of the high-throughput sequencing data, we confirmed the results by analysis of three miRNAs (hsa-miR-221-3p, hsa-miR-126-3p, and hsa-miR-495-3p) using qRT-PCR. We hope that this approach has ensured the reliability of the data obtained and made it possible to detect true differences in miRNA content in plasma and serum across pregnancy.

## 4. Materials and Methods

### 4.1. Study Participants

For the present study, a total of 42 serum and plasma blood samples from seven women across pregnancy (at three points—first (8–13 weeks), second (18–25 weeks), and third (30–35 weeks) trimesters) were collected. The women included in the study had normal singleton pregnancies, with no obstetric or gynecological abnormalities (such as preeclampsia, gestational diabetes, pregnancy-induced hypertension, or intrapartum infection) at any time in pregnancy. Patients with chronic diseases (hypertension, cardiovascular disease, renal disease, hepatitis, or diabetes) or with gestational diseases were excluded from the study.

The study was approved by the Institutional Review Board of the D.O. Ott Research Institute of Obstetrics Gynecology and Reproductology (St. Petersburg, Russia), No. 97 from 27.06.2019. Informed consent was signed by all women prior to their inclusion in the study and to processing of their personal and medical data. The study was performed in accordance with the Declaration of Helsinki.

The study workflow is presented in Figure 1 of Additional File 3.

### 4.2. Sample Preparation

Blood samples were collected in Improvacuter tubes without anticoagulant (for serum) and in Improvacuter tubes with K2EDTA (all purchased from Guangzhou Improve Medical Instruments Co. Ltd., Guangzhou, China) (for plasma). For the serum samples, the blood was allowed to clot by leaving it undisturbed at room temperature for 30 min following collection of the whole blood, following which the clot was removed by centrifugation at 3000× *g* for 20 min at 4 °C. For the plasma samples, tubes with blood were centrifuged at 1500× *g* for 10 minutes at 4 ℃. The supernatant was carefully transferred to a sterile tube and centrifuged again at 2500× *g* for 10 min at 4 °C in order to pellet any debris and insoluble components. After the centrifugation steps, serum or plasma samples were immediately aliquoted in cryotubes, frozen, and stored at −80 °C until further processing. All procedures with serum and plasma were done in parallel and at the same time.

### 4.3. Small RNA Isolation and Library Preparation for Sequencing

Small RNA was extracted from 200 μL plasma or serum samples using miRNeasy Serum/Plasma Kit (Qiagen, Germany), according to the manufacturer’s instructions, and the RNA was resuspended in 12 μL of RNase-free water and then stored at −80 °C until library preparation.

Small RNA libraries were prepared using the QIAseq miRNA Library Kit (Qiagen, Germany) according to the manufacturer’s protocol. Briefly, RNA samples were ligated with 3′ and 5′ adapters. After ligation, reverse transcription was done. The cDNA was purified using magnetic beads, then eluted with 17 µL nuclease-free water. The cDNA samples were used as templates for subsequent PCR. The samples were barcoded by unique indexes during the PCR amplification to allow pooling of libraries before sequencing. The PCR products were cleaned up using the magnetic beads and eluted with 25 µL nuclease-free water. For assessment of the yield, size distribution, and molar concentration of the amplified DNA libraries, the samples were run on 2200 Tapestation Instrument with High Sensitivity D1K ScreenTape and High Sensitivity D1K Reagents (all purchased from Agilent Technologies, Santa Clara, CA, USA). The quantity of libraries required for sequencing was determined according to the manufacturer’s protocol using the miRNeasy Serum/Plasma Kit (Qiagen, Germany).

### 4.4. Illumina Sequencing

Libraries were sequenced on a HiSeq 2500 (Illumina, San Diego, CA, USA) with single-end 75 bp reads according to the manufacturer’s protocol.

### 4.5. Clinical Data Analysis

Clinical data were analyzed using Statistica 10.0 (StatSoft, Inc., Tulsa, OK, USA). Continuous variables were presented as the mean ± standard error.

### 4.6. Sequencing Data Analysis

Small RNaseq data were processed, including adaptor trimming and mapping to miRBase to yield raw count data, using the web-based tools of the GeneGlobe Data Analysis Center (https://geneglobe.qiagen.com/us/analyze, accessed on 1 October 2021). Average of the number of high-throughput sequencing reads per RNA category in plasma and serum of pregnant women was calculated per group (n = 7 samples per group). The differential expression was performed on the resulting raw count matrix of 42 individual samples using DESeq2 R package [43] using complex design including batch compensation for paired samples and trimesters. miRNAs were considered to be differentially expressed with the adjusted *p*-value < 0.05 and absolute value of log_2_(fold change) > 1.5. miRDB target prediction database v.6.0 was used to predict miRNA targets [44]. The target genes were considered to be true predictions with the prediction score > 80. Gene enrichment analysis was performed using ClusterProfiler R package [45], the cutoff values were set to pvalueCutoff = 0.05 and qvalueCutoff = 0.01. 

### 4.7. qRT-PCR

The identical RNA samples that were used for NGS analysis were used for results verification by RT-qPCR. cDNAwas synthesized using the miRCURY RT Kit (Qiagen, Germany). Reaction conditions were set according to recommendations by the manufacturer. In total, 2 µL of total RNA were used per 10 µL reverse transcription (RT) reaction. To monitor RT efficiency and presence of impurities with inhibitory activity, a synthetic RNA spike-in (UniSp6) was added to the RT reaction. Validated LNA-enhanced forward and reverse miRCURY primer assays for all targets, including spike-in controls, were obtained from Qiagen. PCR amplification was performed in a 96-well plate format in a Applied Biosystems 7500 instrument (Applied Biosystems, Waltham, MA, USA) using miRCURY SYBR^®^ Green mix (Qiagen, Germany) with the following settings: 95 °C for 10 min, 45 cycles of 95 °C for 10 s, and 60 °C for 60 s, followed by melting curve analysis. To calculate the cycle of quantification values (Cq-values), the second derivative method was used. Cq-values were normalized to the RNA spike-in control level, by subtracting the individual miRNA Cq-value from the RNA Spike-In Cq, thus obtaining delta-Cq (ΔCq) values that were used for the analysis.

## 5. Conclusions

Although the number of microRNA studies during gestation is increasing, most of them are devoted to the search for biomarkers of pregnancy pathologies using one sample type. While not less, and perhaps even more important is the study of the miRNAs role in the progression of normal pregnancy, which was the subject of our pilot research. Our results confirmed that miRNA concentrations and profiles in plasma and serum change across the normal pregnancy. Changes in levels of 10 miRNAs (hsa-miR-7853-5p, miR-203a-5p, hsa-miR-4435, hsa-miR-340-5p, hsa-miR-4417, hsa-miR-1266-5p, hsa-miR-4494, hsa-miR-134-3p, hsa-miR-5008-5p, and hsa-miR-6756-5p) in the plasma or serum samples from pregnant women across gestation were revealed in this study for the first time; however, the findings require further investigation of the particular roles of these miRNAs in pregnancy. Among miRNAs deregulated during pregnancy, four (hsa-miR-200c-3p, hsa-miR-495-3p, hsa-miR-4435, and hsa-miR-340-5p) have been previously reported to be associated with common gestational complications, indicating their crucial role in pregnancy. We believe that our results might help set the basis for further investigation of molecular mechanisms involved in regulation of pregnancy and the search for biomarkers of severe gestation abnormalities, such as preeclampsia, gestational diabetes mellitus, and preterm birth, using miRNA high-throughput sequencing. Thus, our future plans include collection, sequencing, and analysis of blood samples with pathology. In addition the miRNA profiles were different between plasma and serum, which should be taken into consideration when comparing the results of studies performed using different biotypes. We propose that our findings allow researchers to form a baseline expression profile of normal pregnancy that will help the understanding of how the potential biomarkers of complications change during pregnancy progression. Even though additional research might be needed in order to expand the sample selection including participants of different ethnical background, we can assume that the future pregnancy complications prediction model is highly likely to be based on the detected miRNA level changes in the first and third trimester in both biofluids. 

## Figures and Tables

**Table 1:** Characteristics summary of the pregnant women included in the study.

Characteristic	Research Group (n = 7)
**Maternal Characteristics**	
Age, years	32.71 ± 1.98
Ethnicity	Russian, 7/7
Weight, kg	65.43 ± 2.70
Height, m	1.67 ± 0.03
BMI, kg/m^2^	23.53 ± 0.79
**Gestational Age of Sampling**	
First trimester, weeks	11.07 ± 0.41
Second trimester, weeks	21.77 ± 1.11
Third trimester, weeks	32.54 ± 0.89
**Pregnancy Outcome**	
Mode of delivery	Vaginal delivery/Cesarean section, 6/1
Gestational age at delivery, weeks	39.90 ± 0.35
Fetal weight, g	3481.43 ± 125.12
Fetal height, cm	51.57 ± 0.57

Continuous variables are presented as the mean ± standard error. BMI, body mass index. All women had no chronic diseases and had normal singleton pregnancies, with no gestational abnormalities. The paired serum and plasma blood samples were collected from each woman at three gestational ages. Gestational age was calculated by ultrasound dating conducted during enrolment. Caesarean section was performed in one case for a woman with a breech presentation of the fetus.

**Table 2:** The 36 miRNAs with the significant level differences between plasma and serum samples taken from the pregnant women without gestational complications (*p*-value adjusted to multiple comparisons < 0.05).

Upregulated in Plasma Compared with Serum			Downregulated in Plasma Compared with Serum		
miRNA	log2 (fold change)	Adjusted *p*-value	miRNA	log2 (fold change)	Adjusted *p*-value
hsa-miR-195-5p	2.1037	4.173E-05	hsa-miR-130b-5p	−1.9032	0.0023
hsa-miR-103a-3p	1.9215	0.0138	hsa-miR-4513	−2.1794	0.0062
hsa-miR-103b	1.8966	0.0159	hsa-miR-134-3p	−1.7922	0.0090
hsa-miR-20a-5p	1.8640	0.0024	hsa-miR-6811-3p	−2.2019	0.0090
hsa-miR-101-3p	1.8614	0.0004	hsa-miR-6780b-5p	−1.6910	0.0104
hsa-miR-26a-5p	1.8337	0.0022	hsa-miR-5698	−3.0858	0.0125
hsa-miR-126-3p	1.8255	0.0090	hsa-miR-4742-5p	−2.0452	0.0149
hsa-miR-16-5p	1.8214	0.0164	hsa-miR-138-5p	−2.1160	0.0149
hsa-miR-451a	1.8079	0.0279	hsa-miR-6756-5p	−2.3664	0.0159
hsa-miR-320a	1.7348	0.0390	hsa-miR-3151-5p	−2.1801	0.0180
hsa-miR-221-3p	1.6173	0.0390	hsa-miR-7152-5p	−2.2668	0.0215
hsa-let-7d-5p	1.6133	0.0245	hsa-miR-571	−1.6354	0.0249
hsa-miR-144-3p	1.5750	0.0125	hsa-miR-4650-5p	−1.5487	0.0304
hsa-miR-32-5p	1.5547	0.0225	hsa-miR-5008-5p	−2.2514	0.0305
hsa-miR-320b	1.5117	0.0390	hsa-miR-8054	−1.6772	0.0367
			hsa-miR-1266-5p	−1.5223	0.0390
			hsa-miR-181a-3p	−1.8287	0.0390
			hsa-miR-3689d	−1.6338	0.0420
			hsa-miR-6754-3p	−1.6791	0.0429
			hsa-miR-6748-5p	−2.1050	0.0500
			hsa-miR-518c-3p	−1.7148	0.0496

Adjusted *p*-value and log2 (fold change) of plasma versus serum. *p*-value adjusted for multiple comparisons using the Benjamin–Hochberg method.

**Table 3:** The top 15 enriched GO terms across predicted targets of miRNAs with different levels in plasma and serum.

GO ID	GO Term	Gene Number for Term	Adjusted *p*-Value
GO:0003712	Transcription coregulator activity	208	2.192646 × 10^−14^
GO:0000987	Proximal promoter sequence-specific DNA binding	193	2.831005 × 10^−14^
GO:0004674	Protein serine/threonine kinase activity	155	1.493812 × 10^−10^
GO:0001228	DNA-binding transcription activator activity, RNA polymerase II-specific	160	3.029877 × 10^−10^
GO:0019199	Transmembrane receptor protein kinase activity	39	3.064696 × 10^−7^
GO:0051020	GTPase binding	160	5.003948 × 10^−7^
GO:0046332	SMAD binding	40	2.917633 × 10^−6^
GO:0019003	GDP binding	33	2.894027 × 10^−5^
GO:0003682	Chromatin binding	155	3.486248 × 10^−5^
GO:0019787	Ubiquitin-like protein transferase activity	121	6.411654 × 10^−5^
GO:0003924	GTPase activity	88	8.003687 × 10^−5^
GO:0008013	Beta-catenin binding	39	8.173702 × 10^−5^
GO:0050839	Cell adhesion molecule binding	153	1.619180 × 10^−4^
GO:0035091	Phosphatidylinositol binding	84	1.799412 × 10^−4^
GO:0032550	Purine ribonucleoside binding	115	1.799412 × 10^−4^

GO, gene ontology.

**Table 4:** The top 15 enriched KEGG pathways across predicted targets of miRNAs with different levels in plasma and serum.

KEGG ID	KEGG Term	Gene Number for Term	Adjusted *p*-Value
hsa04151	PI3K-Akt signaling pathway	143	8.615448 × 10^−12^
hsa04010	MAPK signaling pathway	129	8.204628 × 10^−14^
hsa05165	Human papillomavirus infection	120	6.230594 × 10^−7^
hsa04144	Endocytosis	98	1.338059 × 10^−7^
hsa05205	Proteoglycans in cancer	96	1.371966 × 10^−12^
hsa04360	Axon guidance	93	3.946275 × 10^−15^
hsa04810	Regulation of actin cytoskeleton	92	3.863385 × 10^−9^
hsa04014	Ras signaling pathway	92	1.404485 × 10^−7^
hsa04510	Focal adhesion	88	9.398793 × 10^−10^
hsa05163	Human cytomegalovirus infection	86	2.219365 × 10^−6^
hsa04015	Rap1 signaling pathway	82	1.411583 × 10^−6^
hsa04020	Calcium signaling pathway	82	3.687535 × 10^−4^
hsa04024	cAMP signaling pathway	81	1.014159 × 10^−5^
hsa05166	Human T-cell leukemia virus 1 infection	78	1.141134 × 10^−4^
hsa05131	Shigellosisr	78	5.208530 × 10^−3^

KEGG, Kyoto Encyclopedia of Genes and Genomes.

## Supplementary Materials

The following are available online at https://www.mdpi.com/article/10.3390/life11101055/s1, Additional file 1: Table S1. The number of reads that mapped to miRNA genes for plasma samples taken from women without gestation complications at first trimester of pregnancy; Table S2. The number of reads that mapped to miRNA genes for plasma samples taken from women without gestation complications at second trimester of pregnancy; Table S3. The number of reads that mapped to miRNA genes for plasma samples taken from women without gestation complications at third trimester of pregnancy. Additional file 2: Table S4. The number of reads that mapped to miRNA genes for serum samples taken from women without gestation complications at first trimester of pregnancy; Table S5. The number of reads that mapped to miRNA genes for serum samples taken from women without gestation complications at second trimester of pregnancy; Table S6. The number of reads that mapped to miRNA genes for serum samples taken from women without gestation complications at third trimester of pregnancy. Additional file 3: Figure 1. The study workflow.
