# Supplementary figures and images for: High-Throughput Sequencing of Circulating MicroRNAs in Plasma and Serum during Pregnancy Progression
Source: Life (Basel). 2021 Oct 8;11(10):1055. doi: 10.3390/life11101055 (PMC8539647; doi:10.3390/life11101055)

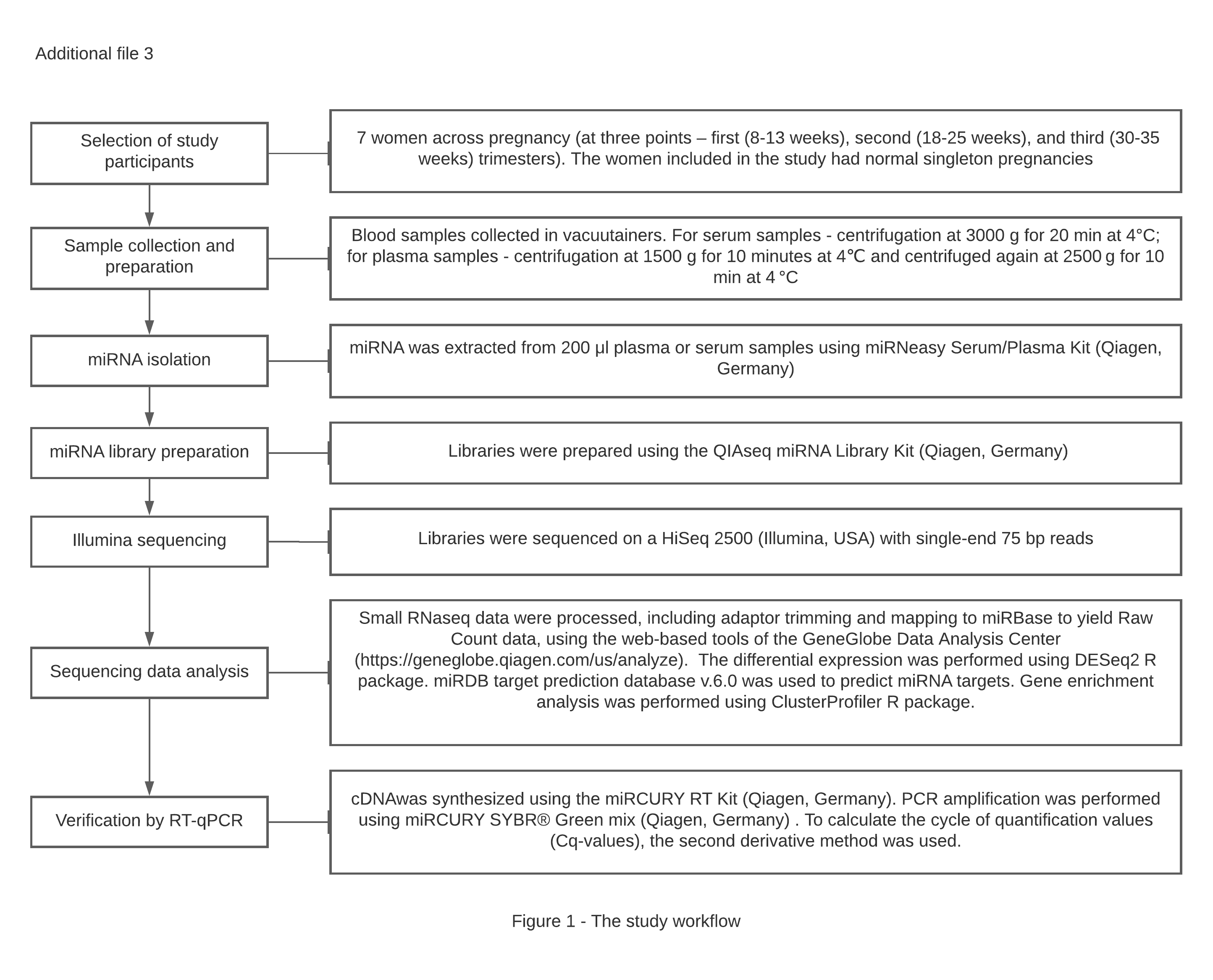

Supplement: Supplementary file 1 [file life-11-01055-s001.zip › Additional file 3.png]
